# Infantile Scimitar Syndrome With Contralateral Pulmonary Vein Stenosis and Refractory Pulmonary Hypertension

**DOI:** 10.7759/cureus.17203

**Published:** 2021-08-15

**Authors:** Janelle Buysse, Ramya Deepthi Billa, Daniel McLennan, Ravi Ashwath, Aditya Badheka, Madhuradhar Chegondi

**Affiliations:** 1 Pediatric Cardiology, University of Iowa Stead Family Children's Hospital, Iowa City, USA; 2 Division of Critical Care Medicine, University of Iowa Stead Family Children's Hospital, Iowa City, USA; 3 Pediatrics, University of Iowa Hospitals and Clinics, Iowa City, USA; 4 Pediatrics, University of Iowa Stead Family Children's Hospital, Iowa City, USA

**Keywords:** scimitar syndrome, pulmonary vein stenosis, pulmonary hypertension, infantile scimitar syndrome, cardiac catheterization

## Abstract

Infantile scimitar syndrome is associated with pulmonary hypertension which can be difficult to manage. We present a three-month-old infant with scimitar syndrome, who eventually developed refractory pulmonary hypertension, posing a significant management challenge. Further workup demonstrated contralateral pulmonary vein stenosis, which is rarely described in scimitar syndrome. Our index case highlights the importance of follow-up cardiac catheterizations in these patients with severe pulmonary hypertension.

## Introduction

This article was previously presented as a meeting abstract at the Society for Critical Care Medicine Annual Conference in February 2021.

Scimitar syndrome is a rare congenital cardiac defect characterized by anomalous pulmonary venous drainage from the right lung to the inferior vena cava [1]. The infantile form of scimitar syndrome presents with severe symptoms, including pulmonary hypertension, and has a high risk of mortality compared to the adult type [1]. Though scimitar vein obstruction is common in these patients, contralateral pulmonary vein stenosis (PVS) is rarely described [2].

## Case presentation

History of presentation

A three-month-old female with a history of scimitar syndrome presented to the emergency department for evaluation of increased work of breathing. In the emergency department, she was started on oxygen via nasal cannula and transferred to our pediatric cardiac intensive care unit (PCICU). On arrival to our PCICU, her oxygen saturation was 85% with tachypnea, intercostal retractions and clear breath sounds on auscultation. Her respiratory support was escalated to high-flow nasal cannula (HFNC).

Past medical history

She had been diagnosed with scimitar syndrome during workup for tachypnea and persistent oxygen desaturation. She was found to have stenosis of the scimitar vein and underwent balloon angioplasty. Following the procedure, she recovered quickly and was discharged home.

Investigations

**Figure 1:**
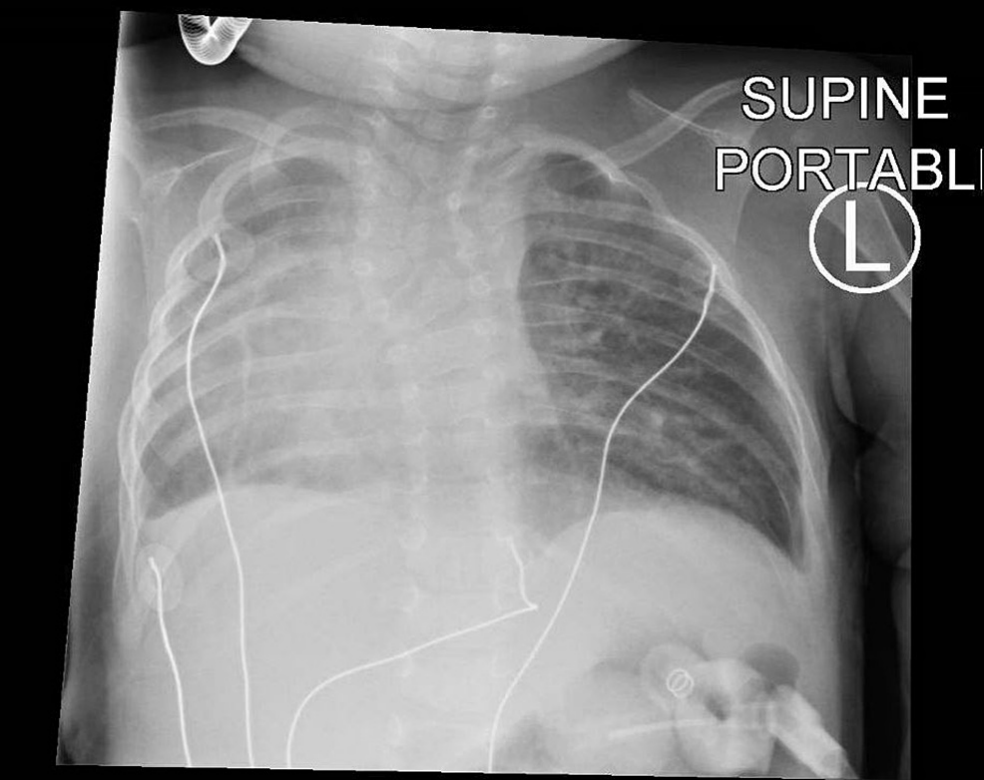
Initial chest X-ray demonstrating right pulmonary hypoplasia, dextroposition, and scoliosis

Chest X-ray demonstrated right pulmonary hypoplasia with dextroposition and scoliosis (Figure 1).

**Figure 2:**
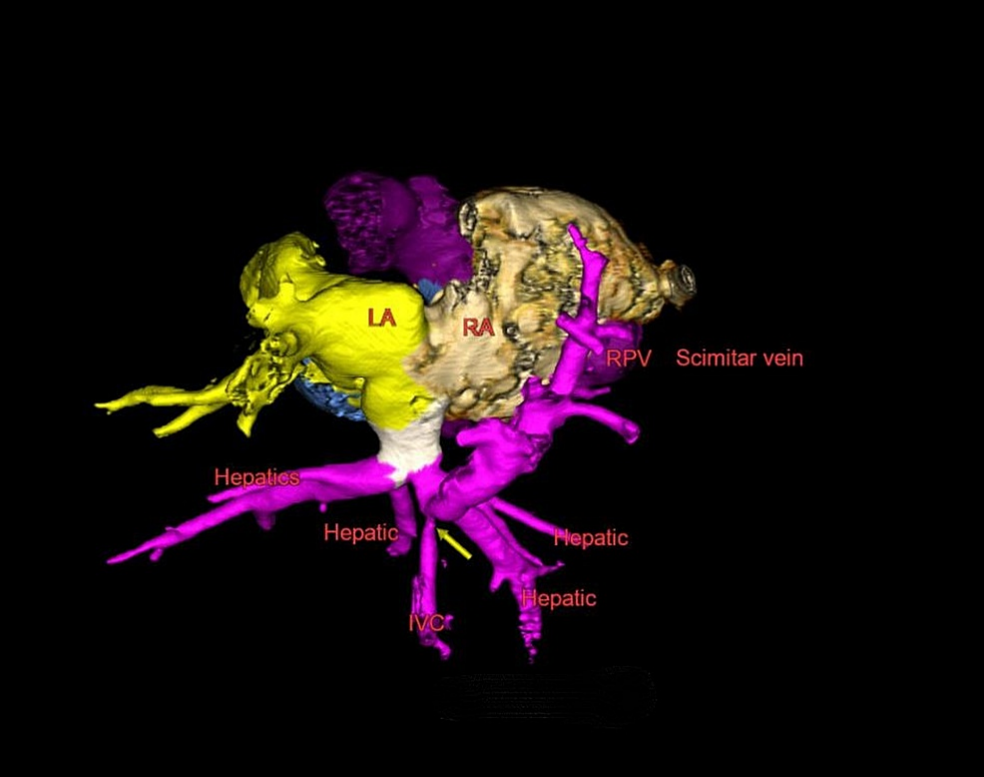
3D reconstruction of CTA images of scimitar vein and IVC demonstrating stenosis of scimitar vein at junction with right hepatic vein CTA = computed tomography angiogram, IVC = inferior vena cava, LA = left atrium, RA = right atrium, RPV = right pulmonary vein; Yellow arrow indicates area of stenosis.

A transthoracic echocardiogram (TTE) revealed dextroversion with partial anomalous venous return drainage of all the right pulmonary veins to the infra-diaphragmatic right hepatic vein. There was a mean gradient of 12mmHg across the entrance of this vein. There was an atrial septal defect (ASD) with bidirectional shunt. The right ventricle was dilated with normal systolic function with flat interventricular septum during systole, indicating at least half systemic right ventricular pressure. Computed tomography angiography (CTA) revealed a scimitar vein draining into the right hepatic vein with an area of severe stenosis measuring 1.1mm (Figure 2).

**Figure 3:**
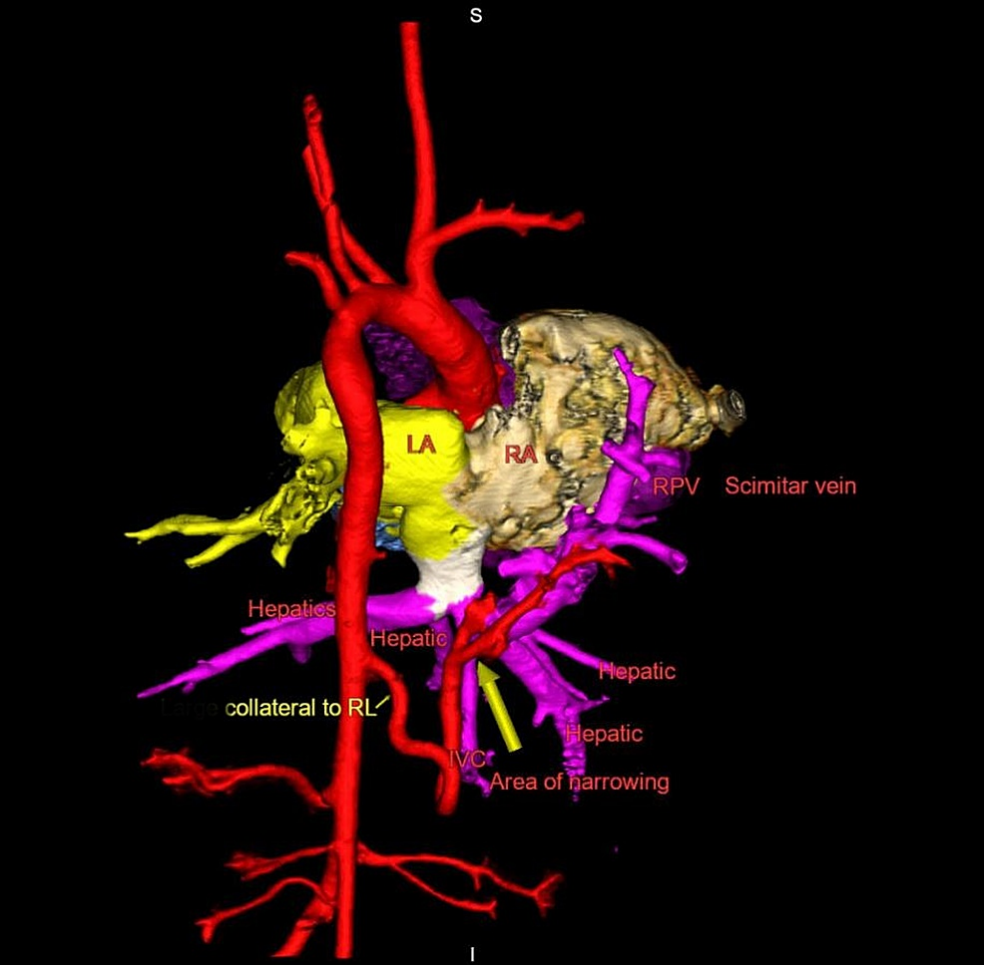
3D reconstruction of CTA images of scimitar vein, IVC, and aorta demonstrating stenosis of scimitar vein at junction with right hepatic vein and moderate-sized aortopulmonary collateral arising from descending aorta and supplying right lower lobe CTA = computed tomography angiogram, IVC = inferior vena cava, LA = left atrium, RA = right atrium, RPV = right pulmonary vein, RL = right lung.

There was a moderate-sized aortopulmonary collateral (APC) supplying the right lung (Figure 3).

The left upper and lower pulmonary veins came together into a confluence before draining, unobstructed, into the left atrium with no areas of stenosis seen.

She underwent cardiac catheterization on 60% oxygen, which revealed supra-systemic pulmonary hypertension resulting in net right to left shunt via ASD with significantly elevated indexed pulmonary vascular resistance (PVRi) at 14 WU.m^2^ (normal < 3 WU.m^2^) and pulmonary to systemic blood flow ratio (Qp:Qs) of 0.8:1 (normal 1:1). With escalation to 100% oxygen and the addition of 40 ppm of inhaled nitric oxide (iNO), the pulmonary artery systolic pressure decreased to 80% of systemic, PVRi decreased to 3.7 WU.m^2^, and Qp:Qs increased to 1.3:1. The scimitar vein was stented at the junction with the right hepatic vein, decreasing the gradient across that region from 11mmHg to 4mmHg. A moderate APC arising from the descending aorta supplying the anterior basal segment of right lower lobe was occluded, prior to release of the vascular plug the patient was monitored for several minutes for any decrease in saturations and with the vascular plug in place iNO and fraction of inspired oxygen (FiO2) were both weaned with maintenance of saturations. The left pulmonary artery and pulmonary veins were not evaluated during this study.

Management

She returned to the PCICU, endotracheally intubated and on a mechanical ventilator. She was started on iNO and sildenafil and extubated to HFNC. She initially tolerated weaning off iNO and remained extubated, however, one week after catheterization she developed episodes of desaturation and bradycardia. During one of these episodes, she required re-intubation and the addition of intravenous epoprostenol. She continued to have frequent pulmonary hypertensive crises, 4-6 times per day, requiring escalation of sedation, initiation of paralytic infusion, addition of epinephrine, milrinone infusions, digoxin, and bosentan.

The follow-up serial TTEs revealed persistent supra-systemic pulmonary hypertension with gradual decrease in right ventricular function. Treprostinil infusion was initiated, and the infusion rate gradually escalated over one month, which was tolerated well. When she continued to have pulmonary hypertensive crises despite maximal therapy, we discussed her case with several pulmonary hypertension centers who all agreed that the overall prognosis was poor and had no additional recommendations. Approximately 10 weeks after the previous catheterization, she underwent repeat catheterization, which revealed supra-systemic pulmonary hypertension, PVRi of 20 WU.m^2^ and significantly elevated left lower pulmonary capillary wedge pressure of 42mmHg. Left pulmonary artery angiography demonstrated long segment stenosis of distal left middle and lower pulmonary veins with a decompressing vessel.

The proximal portion of the left middle and lower pulmonary veins at the confluence with the left atrium appeared to be of normal size. Following the left lower pulmonary artery wedge angiogram there was extravasation of contrast and bloody secretions from endotracheal tube concerning for vascular injury secondary to the suprasystemic pulmonary hypertension. A wedge catheter was inflated in the region of extravasation to tamponade the affected area. While the wedge catheter was in place the patient became bradycardic and hypotensive requiring chest compressions. Once the bloody respiratory secretions cleared the wedge catheter was removed and the rhythm returned to sinus and blood pressure improved indicating that the etiology was likely catheter position irritating the sinus node and stretching open the tricuspid valve. The PVS was not amenable to transcatheter or surgical intervention due to the severity and extension into the lung parenchyma. The catheterization findings were discussed with parents who opted for comfort measures, and the patient died the following day.

## Discussion

Our index case highlights contralateral PVS development in infantile scimitar syndrome and its poor outcome associated with severe pulmonary hypertension. Infantile scimitar syndrome is rare, occurring in one to three per 100,000 live births [3]. Literature has been mostly limited to case series and case reports. Among those descriptions of patients with infantile scimitar syndrome, contralateral PVS is rare, and reported cases in the literature describe dismal outcomes [4]. We reviewed reports of scimitar syndrome with contralateral PVS and summarized these in Table 1.

**Table 1:** Review of literature on contralateral pulmonary vein stenosis in infantile scimitar syndrome PVS = Pulmonary vein stenosis; CHD = Congenital heart defect

Study	Years reviewed	Number of scimitar syndrome patients	Number with contralateral PVS	Interventions performed	Outcome of patients with contralateral PVS
Dusenbery et al. [4]	1964-2011	80	5	Baffle scimitar vein (1), Reimplantation of scimitar vein and repair of other CHD (1), Repair of other CHD (1), No surgery (2)	All died
Al Rukban et al. [5]	2000-2011	16	1	Coiling of collateral vessels	Died
Gao et al. [6]	1964-1989	13	1	Left common pulmonary vein balloon angioplasty and ligation of anomalous systemic artery to right lung	Died
Huddleston et al. [2]	1972-1997	12	1	Left pulmonary vein stent placement, bilateral lung transplantation	Survived
Argueta-Morales et al. [7]	2010	1	1	Left lower pulmonary vein stent placement and subsequent sutureless repair	Died
Onalan et al. [8]	2020	1	1	Sutureless repair of the left pulmonary veins, end-to-end anastomosis of the scimitar vein to the right upper pulmonary vein	Survived, tracheostomy and ventilator dependent

Of the 10 reported cases, there were only two survivors: one required bilateral lung transplantation, and the other required tracheostomy and ventilator dependent [2-8]. Most of the reports did not include information on whether the stenotic segment was distal or proximal, however, the cases described by Argueta-Morales et al. and Onalan et al. included a description of the location of the stenosis and in both cases described the stenosis was located proximally [7,8]. Our case is the only reported case of contralateral pulmonary vein stenosis involving the proximal portion.

Pulmonary hypertension is common in infantile scimitar syndrome reported in half to two-thirds of these patients [2,7]. Causes of pulmonary hypertension are multifactorial, primarily due to obstruction of the scimitar or pulmonary veins and muscularization of the pulmonary arterioles in response to excess pulmonary blood flow due to left to right shunt. There are multiple sources of excess pulmonary blood flow in patients with infantile scimitar syndrome, including flow through scimitar vein, presence of APCs, and associated intracardiac shunts [2]. The presence of left to right shunt poses an additional challenge to managing pulmonary hypertension in these infants. Excessive pulmonary vasodilation can increase left to right shunting, increasing excess pulmonary blood flow and worsening the process of muscularization of the pulmonary arterioles [9].

Follow-up catheterization for pediatric patients with pulmonary hypertension is recommended in the setting of clinical worsening 3-12 months after a significant change in therapy [9]. While catheterization was frequently discussed in our patient, the risks of transportation to the catheterization lab in the setting of frequent pulmonary hypertensive crises with hemodynamic compromise were felt to outweigh the potential diagnostic and prognostic benefits. With the escalation of pulmonary vasodilators, sedation, and paralysis, she eventually had a 48-hour period free from acute episodes of instability and was able to be safely transported to the catheterization lab. Despite repeating catheterization within the recommended timeframe [9], contralateral PVS had developed, explaining the refractory nature of her pulmonary hypertension.

## Conclusions

Contralateral PVS has been rarely reported in association with infantile scimitar syndrome. Our case highlights the importance of follow-up imaging particularly comprehensive diagnostic catheterization in patients with severe pulmonary hypertension. Development of contralateral PVS should be considered in scimitar syndrome patients with refractory pulmonary hypertension.
